# Supplementary material for: PTPN11-related Noonan syndrome predisposes to multifocal low-grade CNS tumors harboring FGFR1 variants
Source: J Neurooncol. 2026 Mar 5;177(1):31. doi: 10.1007/s11060-026-05478-7 (PMC12963110; doi:10.1007/s11060-026-05478-7)
Supplement: Supplementary file 7 — Supplementary Material 7 [file 11060_2026_5478_MOESM7_ESM.docx]

***PTPN11*-Related Noonan Syndrome Predisposes to Multifocal Low-Grade CNS Tumors Harboring *FGFR1* Variants**

Gary Kohanbash^1,2^, Scott Ryall^3,4^, Sam E. Gary^1^, Lindsey M. Hoffman^5^, Robert Siddaway^6^, Anne E. Bendel^7^, Karen W. Gripp^8^, Andrew W. Walter^9^, Jordan R. Hansford^10^, Amy A. Smith^11^, Hong Wang^12^, John M. Skaugen^13^, Uri Tabori^14^, Cynthia E. Hawkins^4,6^, Alberto Broniscer^15^

^1^Department of Neurological Surgery, University of Pittsburgh, UPMC Children’s Hospital of Pittsburgh, Pittsburgh, PA.

^2^Department of Immunology, University of Pittsburgh, Pittsburgh, PA.

^3^The Arthur and Sonia Labatt Brain Tumour Research Center, The Hospital for Sick Children, Toronto, Ontario, Canada

^4^Department of Laboratory Medicine and Pathobiology, Faculty of Medicine, University of Toronto, Toronto, Ontario, Canada

^5^Division of Hematology-Oncology, Children’s Hospital of Colorado, Aurora, CO

^6^Department of Paediatric Laboratory Medicine, Division of Pathology, The Hospital for Sick Children, Toronto, Ontario, Canada

^7^Division of Hematology-Oncology, Children’s Minnesota, Minneapolis, MN

^8^Division of Medical Genetics, Nemours Children’s Health, Wilmington, DE

^9^Division of Hematology-Oncology, Nemours Children’s Health, Wilmington, DE

^10^Division of Oncology, the Royal Children’s Hospital, Melbourne Australia

^11^Division of Pediatric Hematology-Oncology, Arnold Palmer Hospital, Orlando, FL

^12^School of Public Health, University of Pittsburgh, Pittsburgh, PA

^13^Department of Pathology, University of Pittsburgh, Pittsburgh, PA

^14^Division of Pediatric Hematology-Oncology, Department of Pediatrics, The Hospital for Sick Children, Toronto, Ontario, Canada

^15^Department of Pediatrics, Division of Pediatric Hematology-Oncology, UPMC Children’s Hospital of Pittsburgh, Pittsburgh, PA

**Running title**: Brain tumors in Noonan syndrome

**Correspondence**:

Alberto Broniscer, M.D., M.S. 32 Academy Street Arlington, MA 02476; Tel: 412 628-2797; email: [albertobroniscer5704@gmail.com](mailto:albertobroniscer5704@gmail.com)

Gary Kohanbash, PhD. UPMC Children's Hospital of Pittsburgh. 7128 Rangos Research Building. 530 45th St. Pittsburgh, PA, 15201. Tel: 412 692-9456. Fax: (412) 692-8906; email: [gary.kohanbash2@chp.edu](mailto:gary.kohanbash2@chp.edu)

**Changes in affiliation:**

1. Scott Ryall, Ph.D. – Instructor in Pathology, Brigham and Women’s Hospital, Boston, MA
2. Lindsey M. Hoffman – Medical director at Johnson & Johnson, Phoenix, AZ
3. Jordan R. Hansford – Faculty of Health and Medical Sciences University of Adelaide, Adelaide, Australia
4. Alberto Broniscer – Senior medical director at Servier Pharmaceuticals, Boston, MA

**Supplementary Material:**

Supplemental Table 1. Priority genes analyzed.

Supplemental Table 2. Samples collected for molecular characterization.

Supplemental Table 3. Distribution of *PTPN11* genotypes.

Supplemental Table 4. Results of DNA methylation analysis.

Supplemental Table 5. Central radiological review.

Supplemental Table 6. Summary of previously published cases.
